# The Emerging Adult with Inflammatory Bowel Disease: Challenges and Recommendations for the Adult Gastroenterologist

**DOI:** 10.1155/2015/260807

**Published:** 2015-05-04

**Authors:** Itishree Trivedi, Laurie Keefer

**Affiliations:** Division of Gastroenterology and Hepatology, Center for Psychosocial Research, Practice and Training in GI, Northwestern University Feinberg School of Medicine, Chicago, IL 60611, USA

## Abstract

Incidence of pediatric inflammatory bowel disease (IBD) is rising. Adult gastroenterologists are seeing increasing numbers of young adults with IBD, a subpopulation with unique needs and challenges that can impair their readiness to thrive in an adult healthcare system. Most adult gastroenterologists might not have the training or resources to address these needs. “Emerging adulthood” is a useful developmental lens through which this group can be studied. With complex disease phenotype and specific concerns of medication side effects and reproductive health, compounded by challenges of geographical and social flux and lack of adequate health insurance, emerging adults with IBD (EAI) are at risk of disrupted care with lack of continuity. Lessons learned from structured healthcare transition process from pediatric to adult services can be applied towards challenges in ongoing care of this population in the adult healthcare system. This paper provides an overview of the challenges in caring for the post transition EAI from the perspective of adult gastroenterologists and offers a checklist of provider and patient skills that enable effective care. This paper discusses the system-based challenges in care provision and search for meaningful patient-oriented outcomes and presents a conceptual model of determinants of continuity of care in this unique population.

## 1. Introduction

Inflammatory bowel diseases (IBD), including ulcerative colitis (UC) and Crohn's disease (CD), affect 1.4 million people in the USA [1] and up to 396/100,000 persons worldwide [2]. Up to 25% of new IBD diagnoses are made in childhood and the incidence of pediatric IBD is rising [3, 4]. Adolescents with IBD now comprise an increasing proportion of the >500,000 adolescents with chronic diseases that transfer to adult care setting annually [5]. The rising incidence of pediatric IBD, compounded by greater disease severity and complexity [6, 7], has resulted in a large number of IBD patients between the ages of 18 and 25 years that need to be seen in adult care settings. Adult gastroenterologists may need additional training and resources to manage the complex care needs of this sub-population. Similarly, young adults with IBD can lack readiness to thrive in the adult healthcare system. Using the developmental framework of emerging adulthood, this group of IBD patients, who are either graduating from pediatric care or are newly diagnosed and in geographical and social flux, can be more comprehensively understood. Emerging adults with IBD may have higher health services utilization given their unique set of disease characteristics, patient-level barriers like poor disease self-management and disrupted care. In this paper, we will provide a unique overview of the challenges in caring for emerging adults with IBD from the perspective of the adult gastroenterologist and recommend specific skills that adult gastroenterologists and emerging adult patients should have for effective care. We will also discuss the concept of successful transition of care from pediatric to adult care as being vital to ensure lasting success of these post-transitional emerging adult patients in the adult healthcare system. Overall this approach of considering emerging adults are a unique sub-population among IBD patients with their specific needs and challenges can be applied toward creating models of care aimed at improving health service utilization and delivery by training adult gastroenterologists, educating patients, and creating relevant patient-oriented outcomes.

## 2. Emerging Adulthood and Outcomes in IBD

### 2.1. Emerging Adulthood

Referred to in the psychosocial literature as emerging adulthood (Table 1), the first period of adult life from age 18 to 25 corresponds to a short but highly unstable period between adolescence and full adulthood. Emerging adulthood is noted mostly in Western countries and is marked by changing roles (e.g., student, worker, and parent), geographical and social flux, and ongoing dependence on caretakers for financial support and decision-making [8]. Emerging adulthood may be prolonged in the setting of chronic diseases. As pediatric IBD patient progress to adulthood, chances of disease-related complications including strictures and fistulae increase [9], worsening disease complications. Despite more complex disease, young adult patients with IBD demonstrate poor self-management including low adherence to medications which is concerning given that the cost of IBD is being increasingly driven by expensive medications especially biologics [10]. Young adults with IBD also have low attendance of ambulatory clinic visits, putting them at risk of disrupted care [11]. In a retrospective analysis of administrative claims for all-cause costs of care and resource utilization in patients with ulcerative colitis compared to those without ulcerative colitis, the all-cause total health care costs were highest for pediatric and adolescent patients with ulcerative colitis [12]. Similar trend of higher economic burden of IBD among young patients emerges when Crohn's disease patients were added in [13]. Young adults with IBD disproportionately use more emergency services than any other adult sub-population of IBD [14]. Therefore, given all this evidence, emerging adults with IBD (EAI) should be looked at as a distinct group within the IBD population as they a unique set of challenges with higher overall economic burden and greater health service utilization.

### 2.2. Health Outcomes of Emerging Adults with IBD

In chronic disease models, poorer health outcomes have been demonstrated in emerging adults being cared for in adult-centered health settings, likely from a combination of factors including the fact that emerging adults struggle to manage the increased levels of autonomy expected from them in adult care settings [15–17]. In a study of liver transplant recipients, less than half of emerging adult patients managed their liver disease independently, made their own appointment, or understood insurance issues [18]. In congenital heart disease and Type 1 diabetes, the rates of hospitalization [19, 20] and lost-to-follow-up [21, 22] were significantly higher in emerging adults. For IBD in particular, there is a relative lack of data assessing medium- and long-term health outcomes and resource utilization in EAIs; however, some recent studies demonstrate a decrease in continuity of care and increase in resource utilization during this phase. In a study of 95 IBD patients who progressed from pediatric to adult care services in Canada, utilization of health services before and after transfer was studied—there were fewer clinic visits and more documented noncompliance in the first year of adult-oriented care compared to those prior to transfer [23]. Using the 1999 US National Health Interview Survey data for trends in health resource utilization in IBD, Longobardi et al. demonstrated an increase in resource utilization with more emergency room visits, hospitalizations, and surgical intervention early (within 5 years) in the course of IBD [24], a period roughly corresponding with the emerging adulthood period for most IBD patients. Using a health insurance claims database from 2000 to 2006, Karve et al. demonstrated a trend towards less frequent utilization of office visits and outpatient visit and higher number of emergency department visits among 16–25-year age group (encompassing the EAI stage) concerning for loss of care continuity [25]. Therefore, there is evidence of increased interrupted care, more emergency health service utilization, and potentially increased healthcare costs among emerging adult subpopulation of IBD. Requiring cost-effective, multidisciplinary complex care coordination, the EAIs present a new paradigm in gastroenterology to study health services and outcomes optimization.

## 3. Healthcare Transition

Healthcare transition (HCT) (Table 1), a graduation of a patient from pediatric to adult services, can be marked with gaps in care that can lead to poor outcomes including increased morbidity and stress [26]. Like in other childhood-onset chronic diseases like cystic fibrosis [27], emerging adulthood in IBD often corresponds to the time after a patient has been transitioned to adult-oriented services and can have vulnerability to failure of HCT with loss of continuity of care, potentially poor clinical outcomes, and lower quality of life [28, 29]. For all pediatric-onset chronic diseases, the concept of “transition readiness” is vital. Best understood in keeping with the goals of Healthy People 2020 [30] initiative that recommendation a step-wise and age-appropriate approach to transition of care, transition readiness reflects a series of skills in the realms of knowledge, information gathering, self-management and decision-making that need to be mastered by a patient in preparation of HCT [28, 29, 31, 32]. When present, transition readiness can influence a patient's ability to thrive in adult-oriented care setting and foster sustained self-efficacy and motivation for healthy self-management behaviors. However, despite consensus statements put forth by most major pediatric organizations in the past decade, outlining the importance of facilitating transitional care for adolescents with special needs [33–36], most young people with chronic diseases are not receiving adequate transitional readiness services [37]. Efforts to optimize transition readiness extend beyond transfer of care to the adult provider [38] and continue as therapeutic relationship forms between the patient and adult provider, treatment plans are reevaluated and possibly adjusted, and new goals and expectations are set. Thus, the healthcare transition period, when focused on the metric of transition readiness, might provide one opportunity to track and ultimately improve the health outcomes in emerging adults.

### 3.1. Structured Transition, Readiness, and the Emerging Adult

Structured HCT programs in IBD have been studied retrospectively and prospectively with respect to short-term outcomes like knowledge and skills, with mostly descriptive data based on opinion or surveys of the major stakeholder (patient, parent, and provider). The effect of these structured HCT-readiness programs on medium- and long-term outcomes like medication adherence, clinic attendance rates, IBD-specific health related quality of life (HRQoL), and hospitalization rates has not been extensively studied [7, 23]. Small studies in France and Netherlands have assessed the HCT model with joint IBD clinic with both adult and pediatric gastroenterologists and have found positive effects on subjective outcome measurements like self-efficacy (Table 2) [39, 40]. Though several different kinds of transitional care initiatives have been implemented through joint clinics, dedicated transition coordinators, educational didactics, and online tools [7, 28, 38–41], there is no ideal model for structured transitional program in IBD. Additionally, the perspective of adult gastroenterologists regarding transitional care is different from the pediatricians view with unique concerns [42]. Focusing on transitional readiness at the pediatric level provides one method of improving outcomes for EAIs. However, transfer from pediatric to adult services is an inevitable process and not all EAIs have access to a structured transitional care program and may have missed out on concentrated efforts to improve their self-management skills. In the USA, a lack of institutional resources has been reported as major barrier to transitional care for many chronic illnesses [43]. For IBD, structured transition readiness programs are not in place at every pediatric gastroenterology program. Though there is no national insurance constraint dictating timing of transfer to adult care services in the USA, there is still no universal consensus among providers about timing of initiation and completion of the HCT process. Additionally, there is a paucity of data to substantiate the effect of transitional readiness programs in IBD on clinical outcomes like adherence, emergency room utilization, and quality of life indicators, especially after the transfer to adult services. Therefore, in addition to focusing on transition readiness, efforts to create structured post transitional adult-oriented support programs for this subpopulation with IBD can provide a major opportunity to improve their health outcomes.

## 4. Unique Needs and Challenges of the Emerging Adult IBD Subpopulation

**Figure 1:**
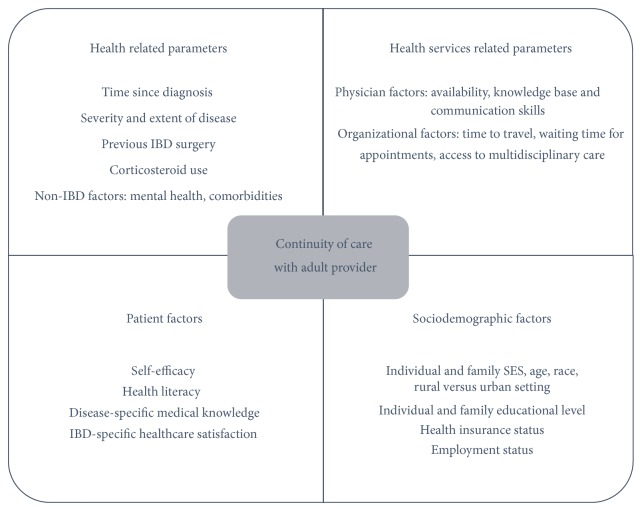
Conceptual model for the care of the emerging adult with IBD. SES: socioeconomic status.

Similar to structured transition from pediatric to adult care, there are no models to our knowledge of structured posttransitional care for the EAI in the adult care world. A conceptual model required to construct a posttransitional emerging adult-specific care program in IBD would require (1) an expanded provider skill set, (2) necessary patient skills for successful and sustained transition, and (3) a consideration of system-based barriers to care provision (Figure 1).

### 4.1. Adult Healthcare Provider Skill Set

In surveys of gastroenterologists about their perception of healthcare transition in IBD, lack of training of adult providers in adolescent medicine is outlined as a major issue in providing care for young adults post-transition [42, 46]. This perceived need for additional healthprovider training to deal with unique issues of young adults is echoed in other chronic disease models like rheumatologic diseases and congenital heart diseases. [47, 48]. Hence, given the unique challenges of EAI, health providers caring for this subpopulation need an expanded skill set, distinct from that required in traditional pediatric or adult practice (Table 2). Knowledge and training in medication adverse effects, sexual health, reproduction and fertility, vocational training and unemployment and complex care coordination, are required.

#### 4.1.1. Understanding Disease Phenotype and Severity

IBD disease phenotype, severity and medical therapies can differ between pediatric and adult patients. Being cognizant of these differences can help adult providers understand the evolution of disease manifestations and treatments for EAIs. Most EAIs will have childhood-onset disease (described as being diagnosed before seventeenth birthday) characterized by greater bowel involvement, progression to extensive colitis, and higher likelihood of requiring immunomodulator therapy [49]. In a retrospective case-control study of 100 adolescents matched with 100 adults, more penetrating Crohn's disease with perianal features and higher incidence of ulcerative pancolitis (67% versus 44%) was noted among adolescents [7]. Growth retardation has been found in up to 88% of children with CD preceding gastrointestinal manifestations [50], with severe linear growth retardation in about 30% of CD patients secondary to undernutrition and inflammation with secondary delayed puberty [51, 52]. With induction of remission either via surgical or biologic therapy, improvement in pubertal progression and skeletal maturation has been noted [53–56]. Additionally, there are increasing incidence and cumulative lifetime exposure of radiation exposure in pediatric IBD [57, 58]. Hence, EAI with childhood-onset IBD presenting to an adult provider may manifest complex disease, growth and nutritional failure and prior excessive exposure to radiation and further care of this patient will depend on nuanced understanding of these factors.

#### 4.1.2. Blending of Pediatric and Adult Care Model for IBD

There are subtle differences in the pediatric and adult model of care in IBD. Pediatric care models for IBD utilize a multidisciplinary approach using close parental involvement with focus on growth, nutritional optimization, and minimizing ionizing radiation. Conversely, traditional adult-oriented approach focuses on the patient as an independent, aware, and motivated consumer of health services and utilizes a shared decision-making model. Adult-oriented care is geared towards symptom control, endoscopic remission, and management of disability including pain while being cognizant of comorbid illness burden and concurrent polypharmacy and cancer surveillance and prevention. An EAI establishing care with adult provided will have graduated from pediatric care and, as such, will be accustomed to a pediatric healthcare environment. Being aware of this and setting clear mutual targets of care will help both adult provider and EIA meet their healthcare goals.

#### 4.1.3. Medication Side-Effects, Sexual and Reproductive Health Issues in Emerging Adulthood

In addition to concerns listed above, there are certain additional medication-related issues that are specifically relevant to EAI. Though beyond the scope of the current review, the increased risk of lymphoma, including hepatosplenic T-cell lymphoma, in young patients on immune-modulator or combined therapy with biologic agents [59], is a major area of concern pertaining to this population. Regarding reproductive health, women with IBD have similar rates of conception as non-IBD patients unless they have undergone surgery (like ileoanal pouch anastomosis [60]). However, the risk of poor pregnancy and perinatal outcomes is increased with active disease at time of conception including spontaneous abortion and low birth weight [61]. Health provider's emphasis on clinical remission during reproductive planning and knowledge of medication safety during pregnancy and the postpartum period is essential. Young adults with chronic illnesses like IBD engage in high risk sexual behavior equally when compared with their healthy counterparts and might have higher rates of sexually transmitted diseases. [62]. Compounding this, sexual health and pregnancy related counseling provided to young females with IBD remain very low [63]. With disease manifestations including diarrhea, fecal incontinence, and perianal disease, treatment side-effects, and surgical procedures with ostomy formation, IBD can have a profound effect on self-image and sexual health of a patient [64]. Therefore, adult provider's competence in knowledge of disease impact on sexual and reproductive health and ability to provide anticipatory counseling are essential in providing appropriate care to this subpopulation.

#### 4.1.4. Unemployment and Disability Issues

The indirect costs of IBD, including cost of work-related opportunity loss, are more than $3.6 billion ($5,228 per person) with active disease associated with lower rates of participation in work force and clinical remission positively associated with probability of employment [24, 65]. There remain deficiencies in employment and financial counseling provided to EAI with only 24% of young adults with chronic illnesses like IBD receive counseling in areas like changing needs with age, how to obtain health insurance, or how to participate in a transition plan in school [66]. Improving work-place retention and minimizing disease-related disability remain a priority in care of EAI. The adult gastroenterologist assuming care of the post-transition EAI must be sensitive to the fact that the patient might not know how to navigate the adult care system, health insurance, and medical prescription systems and may still want parents to be involved in healthcare decision-making. Given these various issues, to assist in the gradual process of complete transition, close and continued coordination between pediatric and adult provider and longer appointment times for initial visits might be needed [32]. Therefore, adult provider training and education in these key areas are needed to reflect the unique needs of EAIs.

### 4.2. Patient Skill Set

A set of patient core-competencies, fostered during transition process and strengthened during posttransition phase in adult setting, can provide EAI with the tools to make health management choices positive (Table 3).

### 4.3. Disease-Specific Knowledge

Fifty-five percent of adult gastroenterologists surveyed in the US considered young adults presenting to adult clinics to have inadequate disease-specific knowledge [46]. In a Canadian study, only 55% of adolescents with IBD could recall when they were diagnosed and less than 25% recalled the location of their disease [41]. In a separate study, among the only 43% of adolescents with IBD who knew their medications, there was very poor knowledge noted about medication side-effects [67]. This is a problem as patient knowledge related to disease and treatments knowledge has been positively linked with important outcomes like health related quality of life (HRQoL) [68, 69]. Hence, the need to measure disease-specific medical knowledge in IBD patients has led to creation and validation of IBD-specific knowledge tests including Crohn's and Colitis Knowledge Score CCKNOW [70] and IBD-KID [71]. These instruments can be adapted for specific use in EAI by including assessment of patient knowledge in issues pertaining to fertility, contraception, medication adverse effects, symptom self-management, and knowledge about insurance and disability management.

### 4.4. Health Literacy

Low health literacy costs the US economy up to $238 billion annually with lower use of preventive services and higher utilization of treatment services including emergency department visits. Adequate health literacy in terms of informational, computational, visual, and computer literacy may be low in EAIs [72] (Table 3). Huang et al. showed that only 36% of patients >18 years age (corresponding with EAI) demonstrated levels of interactive health literacy adequate for readiness to progress to adult-oriented IBD care. As years of education and socioeconomic status do not correlate with it, it is challenging for the care provider to assess an individuals' health literacy and pediatric gastroenterologists can overestimate adolescents' interactive health literacy [73]. Therefore, the adult provider taking over care for EAI must be cognizant of differences in health literacy levels. Age and developmentally appropriate health literacy measures for the EAI can help in accurately identifying and supporting at-risk individuals.

### 4.5. Self-Management

Given the chronic and relapsing nature of IBD, effective self-management behaviors (Table 3) are essential. Though good self-management can be problematic in adults [74, 75], young people are particularly at risk [76]. In a recent study of 67 EAI, most patients thought that self-management behavior improved with advancing age. However, as high as 20% of them still could not perform several self-management tasks [77]. Similar observations were reported previously with only 35% of EAIs (19–21-year age group) scheduling their own clinic visits, 45% ordering medication refills, and 30% reaching out to their provider if problems arose [78]. Hence, challenges with self-management skills continue beyond adolescence into emerging adulthood and targeted interventions in the adult gastroenterology clinics should be undertaken.

### 4.6. Self-Efficacy

The concept of self-efficacy offers great insights into crucial disease self-management skills. Self-efficacy (SE) is a belief in one's ability to organize and execute behaviors necessary to manage challenging situations. SE has been linked to important health outcomes like heart failure hospitalization and mortality in outpatients with congestive heart failure and correlates well with cardiac function [79]. In chronic pain syndromes, level of SE was noted to independently associated with pain-related disability [80]. Based on our previously published adult-specific self-efficacy measure [81, 82], we recently validated an adolescent-specific SE scale for IBD patients [83]. This tool measures self-report across domains of managing daily life, medical care, emotions, and future with IBD. Based on discussion above, EAIs have needs unique compared to both adolescents and adults with IBD; hence, a self-efficacy assessment tool specifically for EAI will allow targeting interventions to specific areas of deficiency in patient's self-motivation and participation in health-sustaining behaviors. Additionally, since EAIs might still be dependent on family for self-management behaviors, including a measure of family/caretaker's knowledge of disease, self-management skills needed and role in EAI care are important.

## 5. System-Based Challenges in the Care of the Emerging Adult with IBD

Lack of universal access to structured pediatric transition readiness program, lack of smooth transfer of patient health information, no standardized age for transfer to adult health services, and lack of health insurance coverage are major system-based challenges in the care of EAI from the perspective of the adult gastroenterologist. Though data about access to structured transition readiness programs in USA is lacking, only 27–60% of pediatric gastroenterology programs in UK and France have a formal transitional program [39]. Smooth transfer of pertinent medical information from a patient's health record and lack of appropriate hand-offs are perceived by gastroenterologists to be a major barrier to successful HCT [42, 84]. The timing of transfer of EAI from pediatric to adult care is not standardized in USA and this adds to challenge of planning a structured model of care for EAI. Though age (18 or 21 years) remains the most widespread reason for transfer to adult care, milestones can be an important trigger for transfer and can add to heterogeneity in age at transfer. For example, in a study of transition practices, varying numbers of practitioners named pregnancy, graduation from high school, and marriage as triggers for transfer [46]. Amongst adult gastroenterologists in the UK, leaving school was the highest-ranking milestone regarding timing of transfer. Pediatric providers ranked disease in remission as most important when deciding timeline of transfer to adult care [42]. Therefore, though certain major milestones in a patient's life can trigger transfer of EAI to adult care, coordination between pediatric and adult providers is essential prior to transfer. In addition to patient preference, impending changes in disease management including surgery, change in treatment plan, and avoidance of times of major social upheaval for the patient should be taken into account for deciding timing of transfer to adult services. With a coordinated and prepared approach to transfer, the EAI arriving in the adult care setting will be best positioned for a successful healthcare relationship with their new adult provider.

In the US, emerging adults remain at high risk for lack of health insurance with age-based termination of health insurance and loss of social support services [5]. Additionally, as described previously, emerging adulthood is marked by instability in terms of employment, higher education, residential status, and financial independence, putting this demographic at additional risk of gaps in insurance coverage. The Affordable Care Act (ACA) of 2010 has extended dependent health coverage for young adults up to the age of 26 and encouraging data trends in coverage for emerging adults are being observed. Data before the initiation of the ACA indicate that as high as 35% of EAs with chronic illnesses had unmet health needs due to excessive cost [85]. Data from the National Health Interview Survey (2008–2012) assessing the impact of the ACA have demonstrated a decrease in percentage of EAs aged 19–25 years who had been uninsured from 9.7% to 6.7%. More EAs have become eligible for coverage through their parents' employment [86]. Compounding the issue of limited patient resources is a lack of institutional resources for young adult programs for IBD including lack of funding and reimbursement and trained support staff. These can directly affect patient access to care making availability of multidisciplinary care and access to social and financial support services difficult which adds to the challenge of effective care provision for the EAI in adult care setting.

## 6. Improving Outcomes in Emerging Adults with IBD: Future Directions

As discussed previously, there is a scarcity of cohort and population-based studies defining the emerging adult patient population in IBD from the perspective of their health service utilization like emergency department utilization, rates of hospitalization and surgery, ambulatory service utilization, medical therapy, and pharmacy costs. Also, there are critical gaps in our understanding of relevant clinical and patient-oriented outcomes for EAI.

### 6.1. Continuity of Care

Continuity of care (CoC) with an adult provider might provide one objective, measurable, actionable, clinically relevant, and patient-oriented outcome in this population. Although personal CoC (care provided by same person over time) is most easily measured, CoC as a concept also includes team continuity (care provision by a team of providers in a medical home) and cross-boundary continuity (care provision in different healthcare settings) [87]. Loss of continuity of care is one of the best described medium-term outcomes after transition in other chronic disease models. In a Canadian study, 40% of patients with Type 1 DM were noted to be lost to follow-up after transfer to adult care [22]. Among young adults with congenital heart defects who transferred to adult care at age 18 due to healthcare regulation changes, 27% had no follow-up appointment since the age of 18 [48]. In a large retrospective study of young adults with DM, physician continuity was a protective factor in DM-related hospitalization with patients maintaining the same physician with a relative risk of 0.23 (95% confidence interval [CI] 0.05–0.79) [20]. More specific to IBD, based on structured interviews with pediatric and adult providers using the Social-Ecological Model of Adolescent and Young Adult Readiness to Transition (SMART) [88], Paine et al. demonstrated that continuity of care with an adult provider was the most commonly used indicator of transitional success, more important than traditional measures like hospitalization rates, quality of life, and steroid use [76]. As understanding of the EAI subpopulation increases, emphasis on identifying the determinants of CoC, ways to meaningfully measure it, and metrics to identify EAIs at high risk of loss of CoC is needed. The use of the electronic medical record and telehealth technologies can make assessment of CoC, identifying at-risk patients, and filling in gaps in care more feasible. Figure 1 presents our proposed conceptual model of the determinants of CoC for the emerging adult with IBD.

In addition to continuity of care, a measure that captures the overall disease experience and burden from a patient perspective will provide another highly meaningful outcome in the EAI population. Previously used in psoriasis, another chronic inflammatory condition, the concept of “cumulative life course impairment (CLCI),” would be highly relevant to the emerging adult stage of IBD. This is a sum total of physical and psychological effects of disease and of the economic and social sequelae of disease [89] and presents a novel paradigm in patient-centered and patient-reported health outcomes research. For IBD, the components and risk factors for CLCI should be studied to understand the summation of physical and psychosocial burden of this disease and be able to tailor treatment options and finite resources and support services to vulnerable patients.

## 7. Summary and Conclusion

The rising incidence of IBD, particularly pediatric IBD, its chronic nature and low mortality, has resulted in a large number of patients between the ages of 18 and 25 that present to adult gastroenterology clinics after transition from pediatric care or at the time of new diagnosis. Health resource utilization increases during this stage. This age group and its unique set of challenges and barriers to healthcare provision can be understood from the developmental framework of emerging adulthood. The health outcomes and needs of this subpopulation are poorly understood. Adult gastroenterologists need to be equipped with an expanded skill set and patients with a set of core-competencies in order for comprehensive healthcare provision to this population. Continuity of care can be a meaningful and measurable outcome to assess quality of healthcare services provided to these emerging after transition to adult care. We proposed a conceptual model outlining the determinants of continuity of care in emerging adults with IBD. This model can be used to inform policy about resource allocation to this field and guide the comprehensive development of post-transition services for EAIs.

## Figures and Tables

**Table 1:** Definitions of frequently used terms.

Adolescent	Period between ages 10 to 19 corresponding to stage of human growth and development after childhood and before adulthood (WHO definition).

Emerging adult	A person between ages of 18 and 25 years in geographical and social flux with changing roles (e.g., student, worker, and parent) and ongoing dependence on caretakers for financial support and decision making [8].

Health care transition	A gradual, purposeful, and patient-centered process that facilitates change in knowledge, attitude, and self-management behaviors in adolescent patients with chronic diseases to foster skills and attitudes required for life as an adult and assuming responsibility for their health [33, 44, 45].

Transfer	The planned movement of patient and their medical records from one provider to another at a distinct point in time.

**Table 2:** Provider skill set (EAI: emerging adult with IBD).

Adult provider skill set for EAI	
Understanding natural history, disease phenotype, complications and treatment options for IBD in EAI	
Appreciating nutrition, growth, and radiation exposure concerns in EAI	
Recognizing the convergence and divergence of traditional pediatric and adult care models in IBD	
“Expanded skill set”:	
(i) Adverse effects of IBD therapies	
(ii) Sexual and reproductive implications of disease, therapies, and well-being in IBD	
(iii) Unemployment and disability issues	
(iv) Negotiating adult healthcare world—health insurance and medical prescription coverage plans	

**Table 3:** Patient core-competencies for success after transition to adult care.

Medical knowledge	Disease-specific knowledge including the following: Personal disease history (diagnosis, extent, location, and prior surgeries) Medication history (including adverse effects) General information on IBD natural history Disease activity and issues of reproductive health Effect of diet, smoking, and use of NSAIDs Importance of preventive health including immunizations

Health literacy	A patient's ability to obtain, process, and understand basic health information in order to do the following: Navigate the healthcare system Access preventive health services Communicate with healthcare providers Analyze risks and benefits of treatment Interpret tests results Mindfully consume available health-related information

Self-management	Health promoting behaviors including the following examples: Recognition of disease flare and extraintestinal manifestations Adherence to complex medication regimen (e.g., self-injectable and rectally administered therapy) Ostomy care Smoking cessation Calling for medication refills Scheduling clinic visits

Self-efficacy	A belief in one's ability to organize and execute behaviors necessary to manage challenging situations

NSAIDs: nonsteroidal anti-inflammatory drugs.
